# Supplementary material for: Multi-Level Control of the ATM/ATR-CHK1 Axis by the Transcription Factor E4F1 in Triple-Negative Breast Cancer
Source: Int J Mol Sci. 2022 Aug 16;23(16):9217. doi: 10.3390/ijms23169217 (PMC9409040; doi:10.3390/ijms23169217)
Supplement: Supplementary file 1 [file ijms-23-09217-s001.zip › Supplementary Figure S1.pdf]

Supplementary Figure S1 Batnini et al.

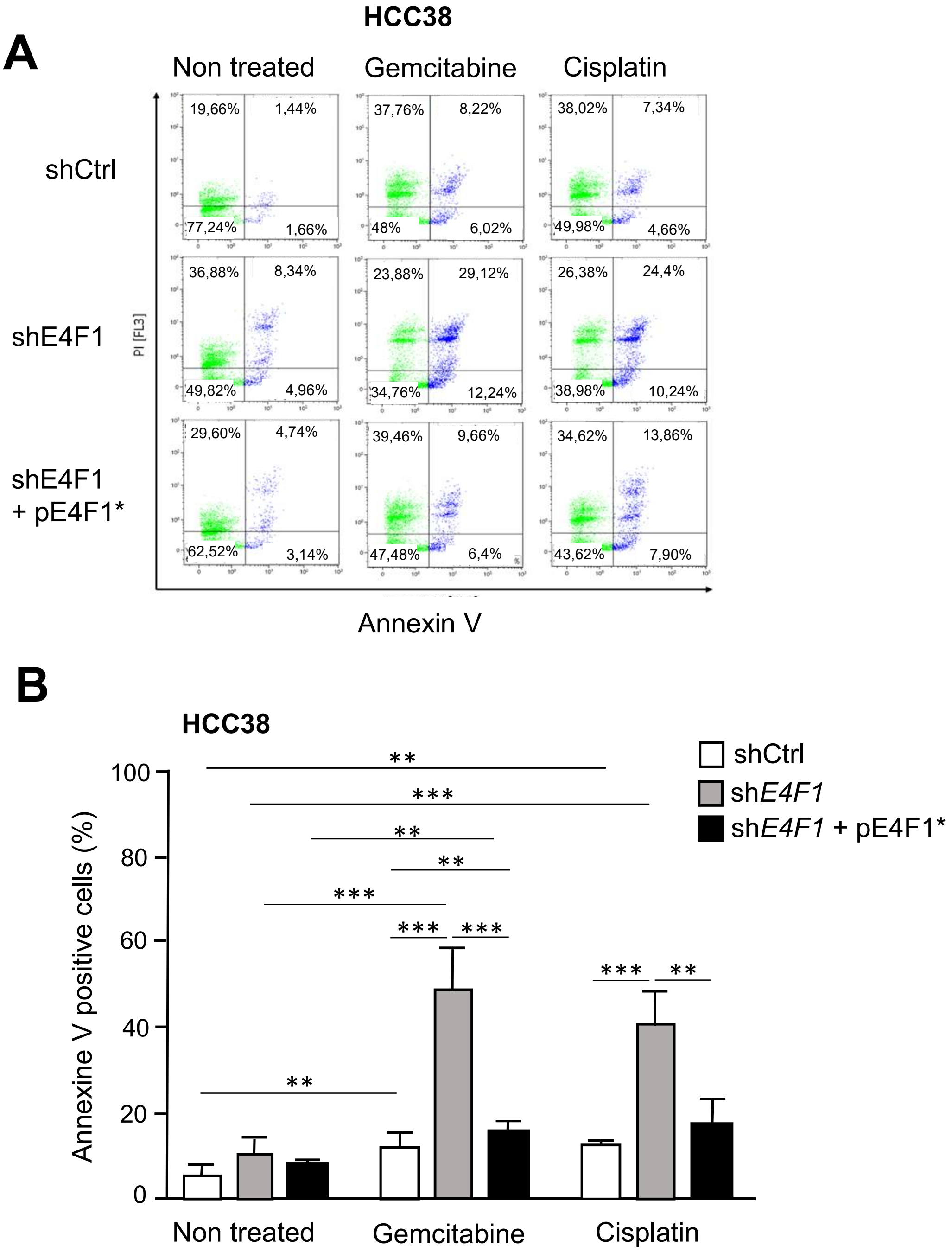

Figure S1: Depletion of the transcription factor E4F1 sensitizes HCC38 Human Triple Negative Breast Cancer (TNBC) cell line to the chemotherapy drugs Gemcitabine and Cisplatin. shRNA-mediated depletion of E4F1 sensitizes TNBC HCC38 cells to chemotherapy drugs (Cytotoxic effect), an effect rescued by the co-expression of the shE4F1-resistant pE4F1* construct. Three days after shCtrl, shE4F1 or shE4F1 + pE4F1* transduction, cells were exposed to sub-lethal doses of Gemcitabine (180 nM, IC30) or Cisplatin (950nM, IC30) for 24 hours and assessed for cell death by) Flow cytometry analysis for apoptosis /necrosis by staining dying cells with annexin V/7-AAD and propidium Iodide (PI). (**A**) Representative flow cytometry profile of annexin V/7-AAD / PI –positive cells, and (**B**) quantitation (FlowJo software) of three independent experiments expressed as mean values ± SD of three independent experiments. ** $p < 0.01$, *** $p < 0.001$ as determined by a one way ANOVA test.
