# Supplementary material for: Multi-Level Control of the ATM/ATR-CHK1 Axis by the Transcription Factor E4F1 in Triple-Negative Breast Cancer
Source: Int J Mol Sci. 2022 Aug 16;23(16):9217. doi: 10.3390/ijms23169217 (PMC9409040; doi:10.3390/ijms23169217)
Supplement: Supplementary file 1 [file ijms-23-09217-s001.zip › Supplementary Figure S2.pdf]

Supplementary Figure S2   Batnini et al.

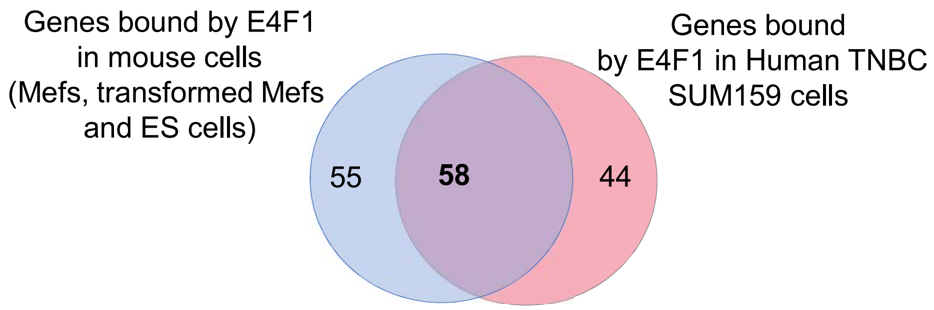

Figure S2: Overlap between all E4F1-bound human genes identified by ChIP-Seq in SUM159 cells and genes bound by E4F1 in mouse cells.
