# Supplementary material for: Multi-Level Control of the ATM/ATR-CHK1 Axis by the Transcription Factor E4F1 in Triple-Negative Breast Cancer
Source: Int J Mol Sci. 2022 Aug 16;23(16):9217. doi: 10.3390/ijms23169217 (PMC9409040; doi:10.3390/ijms23169217)
Supplement: Supplementary file 1 [file ijms-23-09217-s001.zip › Supplementary Figure S3.pdf]

Supplementary Figure S3 Batnini et al.

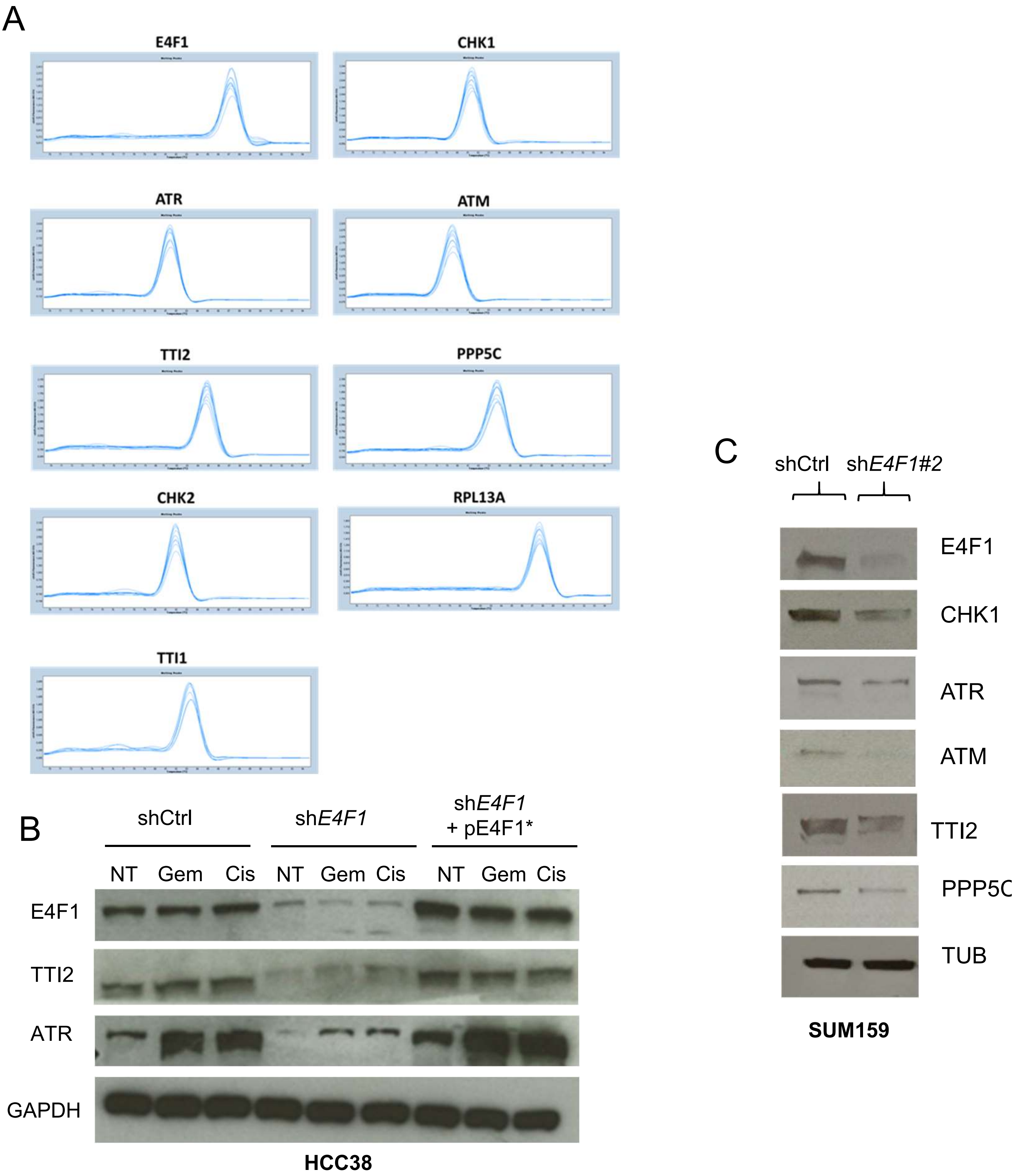

Figure S3: **A-** Melting curve analysis for the RT-qPCR validation experiments in SUM159. **B -** E4F1 depletion also impacts on TTI2 and ATR protein levels in HCC38 TNBC cells: ShRNA–mediated depletion of E4F1 in these cells results in a down-regulation of TTI2 and ATR protein levels in control (NT) as well as in cells treated with Gemcitabine (Gem) or Cisplatin (Cis). This down-regulation is reversed by the co-expression of the shRNA resistant pE4F1* cDNA. **C-** Another shRNA (sh*E4F1#2)* targeting *E4F1* mRNA in SUM159 TNBC cells also impact the protein levels of CHK1, ATR, ATM, TTI2 and PP5C
