# Supplementary material for: Multi-Level Control of the ATM/ATR-CHK1 Axis by the Transcription Factor E4F1 in Triple-Negative Breast Cancer
Source: Int J Mol Sci. 2022 Aug 16;23(16):9217. doi: 10.3390/ijms23169217 (PMC9409040; doi:10.3390/ijms23169217)
Supplement: Supplementary file 1 [file ijms-23-09217-s001.zip › Supplementary Figure S4.pdf]

Supplementary Figure S4 Batnini et al.

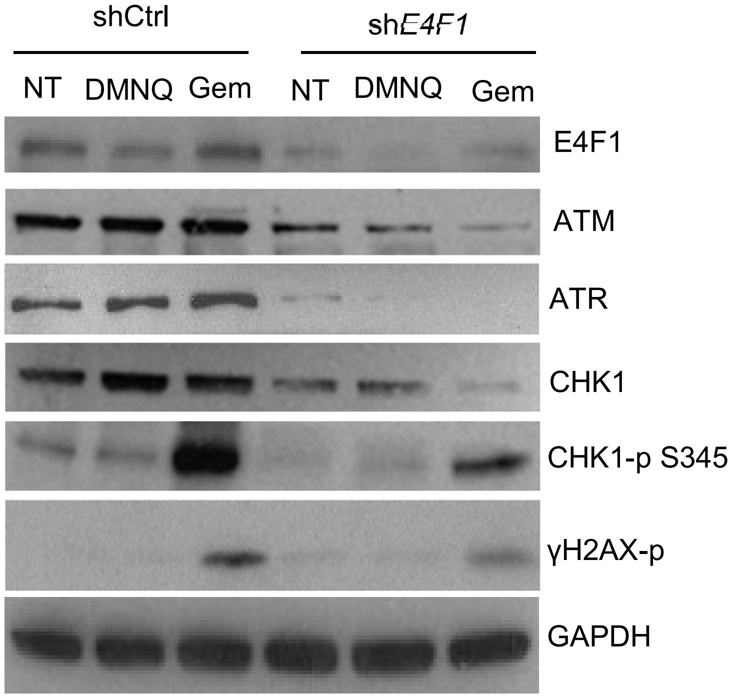

Figure S4: E4F1-depletion in SUM159 impacts on the ATM/ATR-mediated phosphorylation of YH2AX in response to oxidative stress drug, DMNQ and. chemotherapy drug Gemcitabine.
