# Supplementary material for: Multi-Level Control of the ATM/ATR-CHK1 Axis by the Transcription Factor E4F1 in Triple-Negative Breast Cancer
Source: Int J Mol Sci. 2022 Aug 16;23(16):9217. doi: 10.3390/ijms23169217 (PMC9409040; doi:10.3390/ijms23169217)
Supplement: Supplementary file 1 [file ijms-23-09217-s001.zip › Supplementary Figure S5.pdf]

Supplementary Figure S5 Batnini et al.

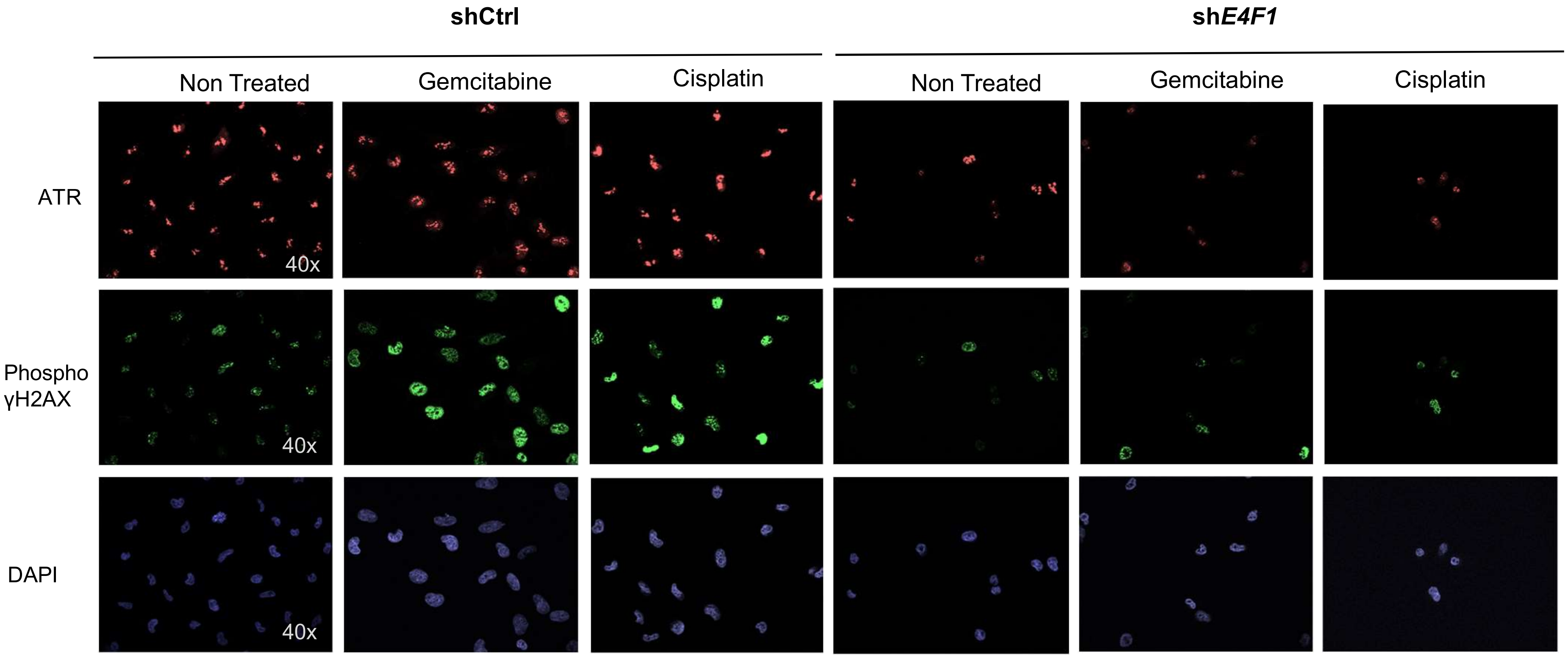

Figure S5: E4F1-depletion impacts on ATM/ATR – CHK1 signalling in response to chemotherapy drugs Gemcitabine and Cisplatin in SUM159 TNBC cells. - Immunofluorescence of DNA (blue) and of phospho S139)- ⊚H2AX (green) and ATR (Red) in SUM159 cells transduced with either control shRNA (shCtrl) or sh*E4F1* and upon antibiotics selection, treated for 24h with Gemcitabine or Cisplatin.
